# The media and cancer: education or entertainment? An ethnographic study of European cancer journalists

**DOI:** 10.3332/ecancer.2014.423

**Published:** 2014-04-17

**Authors:** Ajay Aggarwal, Rekha Batura, Richard Sullivan

**Affiliations:** 1 Institute of Cancer Policy, Kings Health Partners Integrated Cancer Centre, Guy’s NHS Foundation Trust Campus & Kings College London, Department of Research Oncology, Bermondsey Wing, London SE1 9RT, UK; 2 Guys & St Thomas’ NHS Trust, Department of Clinical Oncology, London SE1 7EH, UK; 3 Kings College London, London SE1 9RT, UK

**Keywords:** Cancer, health information, media, public reporting, research, trials

## Abstract

The media plays a vital role in informing the public about new developments in cancer research and influencing cancer policy. This is no easy task, in view of the myriad of trials and wonder drugs that purport to be the ‘magic bullet’. However, misrepresentation can have profound consequences. In this qualitative study, we sought to understand the interaction between the media and cancer through the perspective of European science journalists by defining their attitudes towards current cancer research and challenges faced when reporting science news. A total of 67 respondents took part in this online survey, which was distributed by the European CanCer Organisation (ECCO) to all its media contacts between June and September 2013. Fifty-three per cent had over 20 years experience in reporting science news stories. The respondents utilised a number of media formats, including newsprint, online services, and radio.

Fifty per cent ranked public interest as the greatest influence on their selection of cancer research topics, followed by topicality. Respondents were conscious of being fed ambiguous and exaggerated results from trials by the research community. Sixty-five per cent of respondents would appreciate access to a forum of experts willing to provide comment on new research findings. Seventy per cent highlighted the importance of prompt responses from scientists and researchers during correspondence, and the need to have advance warning of new developments (49%). To conclude – coverage of cancer related issues and scientific advances require greater collaboration between the press and cancer healthcare community to provide both credibility and accountability for the health information disseminated. Key areas include a more precise definition of the research context and differentiation of absolute and relative risks, as well as individual and population risks and an informed discussion about the realities and limitations of cancer care and research.

## Background

In high-income and many emerging economies, the popular media, through a variety of media formats (web, print, television and so on), plays an integral role in influencing the public’s awareness and perception of cancer [1]. The media purport to provide information that is focused, relevant, and easily understandable, enabling the wider public to identify applicable risk factors and adopt healthy lifestyles and choices, as well as promote research into cancer (directly benefiting charitable funders). This, in turn, can modulate the time lag between symptoms to diagnosis and therefore survival rates [2]. However, positivism around the media’s role in cancer has been seriously challenged.

During the last two decades, the media has inundated the public with often contradictory and imbalanced stories regarding cancer [3, 4]. Much of this information has been ambivalent, conflicting, and scientifically questionable, resulting in a public frenzy about cancer [5–7]. This is particularly noticeable with respect to nutrition and diet and the risks conferred by exposure to specific foodstuffs. For example, the overestimation of the protective benefits of vitamins that may, in fact, increase risk [8]. The subsequent confusion amongst the media is then transferred to the public, who may fear the consequences of continuing their current food habits [9].

News coverage may frequently omit limitations of research or the context in which it was conducted. A recent study reviewing media coverage of medical research found that newspapers were more likely to cover observational studies than randomised control trials and preferentially cover research with weaker methodology [10, 11]. However, in mitigation, journalists point to the fact that they have the often onerous task of making esoteric cancer science ‘interesting’ to the general public. Furthermore, they are continually ‘fed’ with stories by both public and private sectors eager, for different reasons, to gain media impact for research findings [12]. In the process, however, much of the science and statistics is lost with omission of key facts, lack of balance or unreasonable emphasis [13].

The media also reports certain cancers more than others, relative to their incidence in the general population. One study in the United States reviewing content analysis of media outlets, including television and print media, found that breast, colon, and brain cancers as well as leukaemias were all over-represented relative to their incidence, while non-Hodgkin’s lymphoma, prostate, and lung cancers were all under-represented [14]. Similar findings have been noted in other studies, with bladder cancer frequently under-represented, and breast cancer dominating coverage about specific cancer sites across all media formats [15–17]. This tends to mirror the degree of celebrity endorsement and corporate sponsorship [15, 18, 19]. As a result, the public overestimate their risks of certain cancers based on their overrepresentation in the media, for instance brain cancers [20]. The suggestion is that too many ‘interest groups’ whether charitable, commercial or academic, fuel media activity, not through a wish for balanced informed communication but for their own partisan interests, rather than a media agenda.

In this qualitative study, we sought to understand the interaction between media and cancer through the perspective of European science journalists and editors experienced in cancer communication through a variety of media formats. The aim was to identify their attitudes towards current cancer research and challenges faced when reporting science news.

## Methods

Between June and September 2013, a survey was carried out as part of the broader Eurocancercomms program looking at cancer communication across Europe. The European CanCer Organisation (ECCO) distributed an online questionnaire to its list of media contacts (classified as health and research writers) in Europe (n = 93). A total of 63 responses were received (67% response rate). Two-third of the subjects were full time staff, with the remainder freelance reporters and editors.

**Figure 1.**
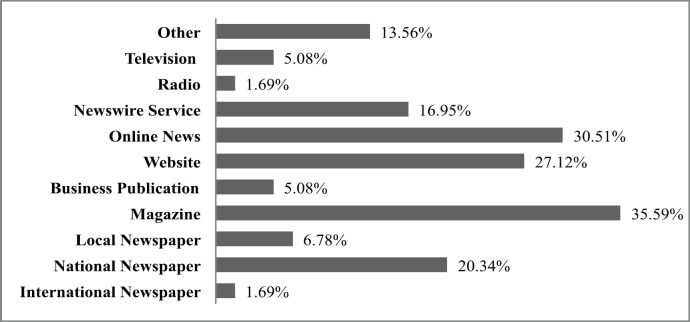
Media output of respondents (% of all respondents).

The majority of respondents were from the United Kingdom (42%) and the United States (19%). The remaining respondents came from Australia, Belgium, Finland, France, Germany, India, Italy, The Netherlands, Spain, Canada, and Asia/Pacific. Respondents in general had significant experience in this area. Fifty-three per cent had greater than 20 years of media experience, 30% 10–20 years, and 17% had less than ten years experience. Respondents were involved in a range of media outputs, with nearly two-third of subjects working for magazines or online news services. Approximately, 30% worked for newspapers covering predominately local and national news (Figure 1). Seventy-one per cent were involved in writing general and/or healthcare articles, 47% science stories related to topical news and events, 31% specific areas of scientific research, 19% general science topics, and 10% science events.

## Results

Respondents were asked to rank what factors influenced their selection of cancer research topics when reporting. Fifty per cent ranked public interest as the greatest influence followed by topicality. On the other end of the scale, industry involvement (32%) and organisational press releases (21%) were ranked as the least influential. The survey also asked respondents to indicate which topics out of a preselected list were more likely to be of greatest interest to their readership/viewers. Topics reflecting patient involvement (personal experience of cancer) dominated (74%), whilst drug development, genetics, cancer prevention, and technology innovation were considered to appeal to readers more than other areas of science.

Respondents were then asked to state the preferences of editors from the same list of preselected topics. As per the previous results patient involvement (personal stories), stories specific to cancer type—particularly breast cancer—and drug development dominated editorial preferences.

The survey explored the complexities and difficulties associated with reporting cancer research. The respondents felt that research findings were overstated by both the media and clinical trials public relations personnel. They were also conscious of being fed ambiguous and exaggerated results, disseminated by the research community (both public and private sector). In addition, the intricacy of technical terminology and preponderance of jargon made it difficult to decipher the real substance of the research. However, the survey was not designed to elicit the degree to which the media ‘community’ acquiesced, and accepted information that they received at face value or the extent to which they directly questioned research findings.

**Figure 2.**
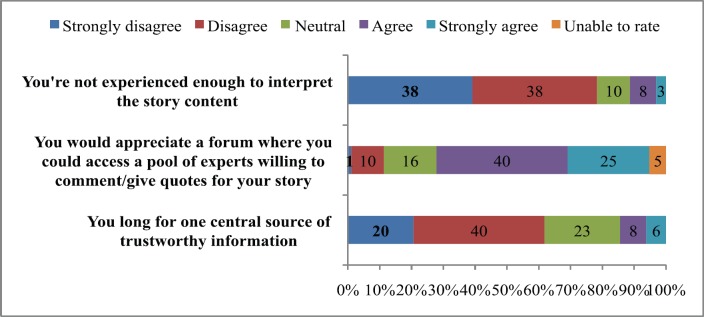
Percentage (%) agreement by respondents to a set of three hypothetical vignettes regarding their experiences as reporters of cancer news.

The respondents to the survey were asked to rate their level of agreement with a set of vignettes which express hypothetical opinions about their experiences as reporters (Figure 2). Sixty-five per cent of respondents agreed/strongly agreed with the idea that they ‘would appreciate access to a forum of experts willing to provide comment on new research findings’. This would be in preference to being able to obtain comprehensive information from a single source; a statement which only 14% of respondents agreed or strongly agreed with.

**Figure 3.**
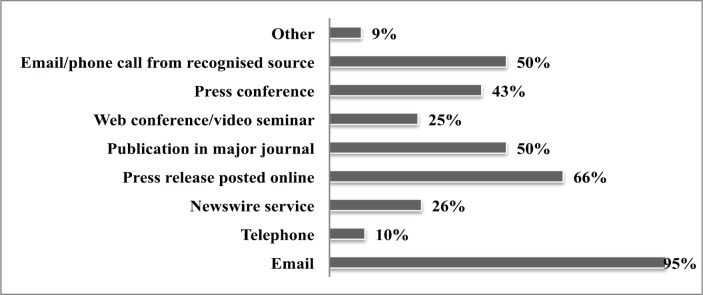
Preferred method of communication of news stories (% of all respondents).

The respondents were asked how they would prefer to receive potential news stories (Figure 3). Whilst primary journal publications are desirable, there remained a preference for electronic and internet-based updates, be they online press releases or e-mail alerts. The respondents were asked to identify their main frustrations when trying to report on health matters and scientific developments. A notable concern was the inconsistent quality and questionable reliability of press releases and information sources. This was exacerbated by delays experienced when attempting to gain clarification from experts in the field resulting in missed deadlines and lost opportunities. In addition, it was hard to distinguish really promising news amidst the plethora of science research findings they are asked to report.

The final two survey questions asked: (i) *what would be respondents’ advice to scientists and researchers? *and (ii) ‘*what one single thing would make the respondents’ lives easier to report cancer stories’? *Similar responses were given to both questions. Seventy per cent reported their most important priority would be to receive prompt responses from scientists and researchers during correspondence. This is backed up by 49% saying that they would like more advance warning of new developments and upcoming news. Respondents also noted that when researchers do respond, they would prefer information delivered in an easy to understand and concise format without jargon.

## Discussion

Media coverage of scientific research plays a major role in shaping public opinion and influencing medical practice and as such has an endless capacity to mislead. Redmond noted that the use of print media and interpersonal sources of health information are most consistently associated with self-reported health behaviours [21]. Another study reviewed media coverage following the introduction of the human papilloma virus (HPV) vaccine in the United States in 2006. There was a significant increase in the news reports on HPV infection and subsequent knowledge on the subject. However, many of the reports missed key information, such as the need for continued cervical screening following vaccination and that HPV is sexually transmitted [22].

There is clearly a schism between the media and cancer research community. This lack of understanding and inability to convey the importance of subject matter to media personnel results in limited coverage of what could be pivotal research work. Instead journal articles are published, which tend to be too technical or over-simplified for public consumption. There is little balance regarding benefits and problems that may be over-hyped or under-stated. The resulting confusion raises serious questions as to whether the media is a suitable vehicle for promoting public understanding of cancer research, or simply an unthinking accomplice of the research community’s need to constantly position itself in the public space. Or, is it simply a reflection of the confusion, ambiguity, and complexity of cancer research?

Spencer reviewed seven year’s (2002–2007) worth of media coverage of the role of environmental pollutants in the aetiology of breast cancer. He found that the news media downplayed and frequently overlooked the evidence. The authors points to a number of reasons for this, including (i) journalist’s lack of awareness about environmental health science, (ii) the seemingly higher standards of proof for research findings that implicate chemicals in disease than for other types of scientific research, (iii) the establishment’s lack of acceptance of environmental theories of breast cancer, and (iv) economic pressures on news outlets not to alienate their advertisers [23]. However, this is not an isolated case. Equally misleading are media stories covering complementary and alternative medicines, with much of the information considered inaccurate, dangerous, and/or incomplete [24].

News reports about novel therapies and expected gains in survival make their way into headlines as soon as a clinical trial is concluded, but are often short on information regarding costs, and adverse effects. Few media stories report the failure rates or quality of life endpoints thus conveying an inaccurate summary of the outcomes and prognosis [25]. Conversely, the media may produce sensationalist reports on drugs that have already been granted a licence for use, offering false hope of a new blockbuster drug becoming available for patients fighting against cancer. This has direct effects on clinician–patient interaction. For example, one of our authors (Ajay Aggarwal) recently engaged with a patient who had brought in a *Daily Mail *(a UK daily newspaper) clipping about docetaxel for prostate cancer as a new drug. Aggarwal had to explain that it was already available on the NHS, which seemed to paradoxically disappoint him. However, as the media would be quick to point out, these positive stories of innovations and ‘breakthroughs’ are fuelled by the press offices of major charities, the academic organisations, and the private sector.

Stryker reported that the ‘newsworthiness’ of medical journal articles predicted their visibility in newspapers. Press releases were an important factor in predicting the extent of newspaper coverage. They found that the reporting of medical breakthroughs that were ‘topical, stratifies risk based on demographic and lifestyle variables, and had lifestyle rather than medical implications’ were covered more widely. Medical journals tended to issue press releases for articles that possess the characteristics journalists are looking for [26]. However, this has had knock on effects, with one study reporting that patients were not satisfied with the information provided, due to media discourse and newsworthiness restricting the reporting of what was actually sought [27].

An increasing trend towards e-media usage amongst the public over the last two decades has made online news the principle source of information on cancer [28]. Media health reporting in this area has increased significantly during the past ten years. A 2006 study examining cancer research stories on the BBC web archive found approximately 260 health stories per year, of which about 170 were classed as relevant to reports of cancer research. The stories focused heavily on breast cancer, followed by lung and prostate cancers, albeit less often than expected in light of their relative prevalence and burden. The area afforded greatest attention was new or improved drugs or vaccines (20% of stories). However, lifestyle choices (12%), genetic developments (9%), and food and drink (8%) also feature prominently [29].

Another study analysed the contents of full-length broadcasts of local television news from a representative sample of the top 50 US media markets (122 stations). It reported that the local television news devoted significant airtime to health stories, yet few newscasts provided useful information. Worryingly, news items with factually incorrect information and potentially dangerous advice were aired [30].

Media coverage of controversial issues is also prone to citing positive publications more often than null scientific publication, and thereby disseminating an inaccurate perception to patients and lay public [31]. Lawrentschuck *et al *[32] characterised the media response to the publication of results from two large randomised prostate cancer screening trials reported in the *New England Journal of Medicine *in 2009, which did not provide conclusive evidence about the benefits of screening [33]. Prospective analysis of the quality and content of print and online media coverage in North America (Canada and the United States), Australasia, and the United Kingdom regarding these trials found conflicting messages. Only 23% of newsprint articles indicated that screening was a positive endeavour, whereas 31% were negative and the remainder were neutral (46%).

The UK media concluded that an inadequate level of PSA (prostate specific antigen) screening was occurring (78% of UK articles), whereas the North American media concluded that PSA screening was excessive (57% of articles in the United States, and 80% in Canada). Worldwide online media reflected US reporting of the trial results [32]. It is, therefore, important that key stakeholders are aware of the impact of media reporting on patient attitudes and preferences with a knock on effect in health seeking behaviour.

The reality is that coverage of cancer related issues and scientific advances requires greater collaboration between the press (journalists and editorial policy setters) and cancer healthcare community to provide credibility to the health information disseminated and more pertinently, accountability [28]. Key areas for improvement include a more precise definition of the context, elucidation of absolute, and relative risks, differentiating between individual and population risks, as well as detailing the history and progression of advances in cancer management and their limitations. Above all it requires an appreciation of the different needs and limitations of the media and the diversity of the general public audience [34].

There is, however, evidence that the media portrayal of research findings is being increasingly challenged. For example, an analysis of the media reporting of the controversial *Lancet Oncology *Commission Delivering Affordable Cancer Care in High Income Countries found that the initial negativism towards the report was rapidly challenged by an online community of commentators and indeed, following the initial media reporting, further commentary was far more balanced. This suggests that the 24 h media cycle and the need to react immediately play heavily against balanced and accurate reporting of cancer [35].

## Conclusion

To conclude—the impact of reporting medical science and specifically cancer has become an important area of public policy. Health care and policy issues germane to the complexities of oncology need to be presented in simple and clear terms and subsequently monitored and managed beyond the routine press briefing. There is a need to preclude the latent hazard of misinterpretation of a publication by one source, which is disseminated by all the channels using that single source for their own story [35]. Pre-existing channels of communication need to be redefined to provide a platform, which encourages informed discussion about the realities of cancer care and research amongst patients, policy makers, advocates, and journalists [36].

## Conflicts of interest

The authors declare that they have no conflicts of interest.

## Author’s contributions

All authors were involved in the conception and design of the manuscript, and performed the initial literature review. All authors revised the manuscript critically for important intellectual content and approved the final version.
